# The Astley Cooper Prize

**Published:** 1862-10

**Authors:** 


THE ASTLEY COOPER PRIZE.—This prize, of 1500 dollars, has been awarded to Dr. Edward Crisp, by the physicians and surgeons of Guy's Hospital, for his essay on the Anatomy, Physiology, and Pathology of the Human Pancreas.

